# Current progress in strategies to profile transcriptomic m^6^A modifications

**DOI:** 10.3389/fcell.2024.1392159

**Published:** 2024-07-11

**Authors:** Yuening Yang, Yanming Lu, Yan Wang, Xianghui Wen, Changhai Qi, Weilan Piao, Hua Jin

**Affiliations:** ^1^ Laboratory of Genetics and Disorders, Key Laboratory of Molecular Medicine and Biotherapy, Aerospace Center Hospital, School of Life Science, Beijing Institute of Technology, Beijing, China; ^2^ Department of Pathology, Aerospace Center Hospital, Beijing, China; ^3^ Advanced Technology Research Institute, Beijing Institute of Technology, Jinan, China

**Keywords:** *N* ^6^-methyladenosine, m^6^A detection, epitranscriptomics, RNA modifications, gene regulation

## Abstract

Various methods have been developed so far for detecting *N* ^6^-methyladenosine (m^6^A). The total m^6^A level or the m^6^A status at individual positions on mRNA can be detected and quantified through some sequencing-independent biochemical methods, such as LC/MS, SCARLET, SELECT, and m^6^A-ELISA. However, the m^6^A-detection techniques relying on high-throughput sequencing have more effectively advanced the understanding about biological significance of m^6^A-containing mRNA and m^6^A pathway at a transcriptomic level over the past decade. Various SGS-based (Second Generation Sequencing-based) methods with different detection principles have been widely employed for this purpose. These principles include m^6^A-enrichment using antibodies, discrimination of m^6^A from unmodified A-base by nucleases, a fusion protein strategy relying on RNA-editing enzymes, and marking m^6^A with chemical/biochemical reactions. Recently, TGS-based (Third Generation Sequencing-based) methods have brought a new trend by direct m^6^A-detection. This review first gives a brief introduction of current knowledge about m^6^A biogenesis and function, and then comprehensively describes m^6^A-profiling strategies including their principles, procedures, and features. This will guide users to pick appropriate methods according to research goals, give insights for developing novel techniques in varying areas, and continue to expand our boundary of knowledge on m^6^A.

## 1 Introduction

In the past decade, it was realized that chemical modifications in internal regions of mRNA and long-noncoding RNA (lncRNA) comprise an important layer of gene regulation (Shi et al., 2019), leading to the emergence of the exciting field of epitranscriptomics. Although the epigenetic code in chromatin is widely accepted, it is still unclear whether RNA possesses similar epitranscriptomic code (Fu and He, 2012). As early as the 1970s, m^6^A modification was found in mRNA and lncRNA of eukaryotes. So far, the top internal base modifications observed in poly(A)-tailed RNAs are m^6^A, m^1^A and m^5^C. Among them, m^6^A is the most widespread one, accounting for 0.2%–0.6% of all adenosines (Desrosiers et al., 1974; Perry et al., 1975). It was reported that m^6^A modification regulates mRNA splicing, translation, degradation, and thus takes part in varying physiological processes such as neural development, cell fate transition, immune response, and DNA damage repair (Patil et al., 2016; Xiang et al., 2017; Wang et al., 2018; Winkler et al., 2019).

**FIGURE 1:**
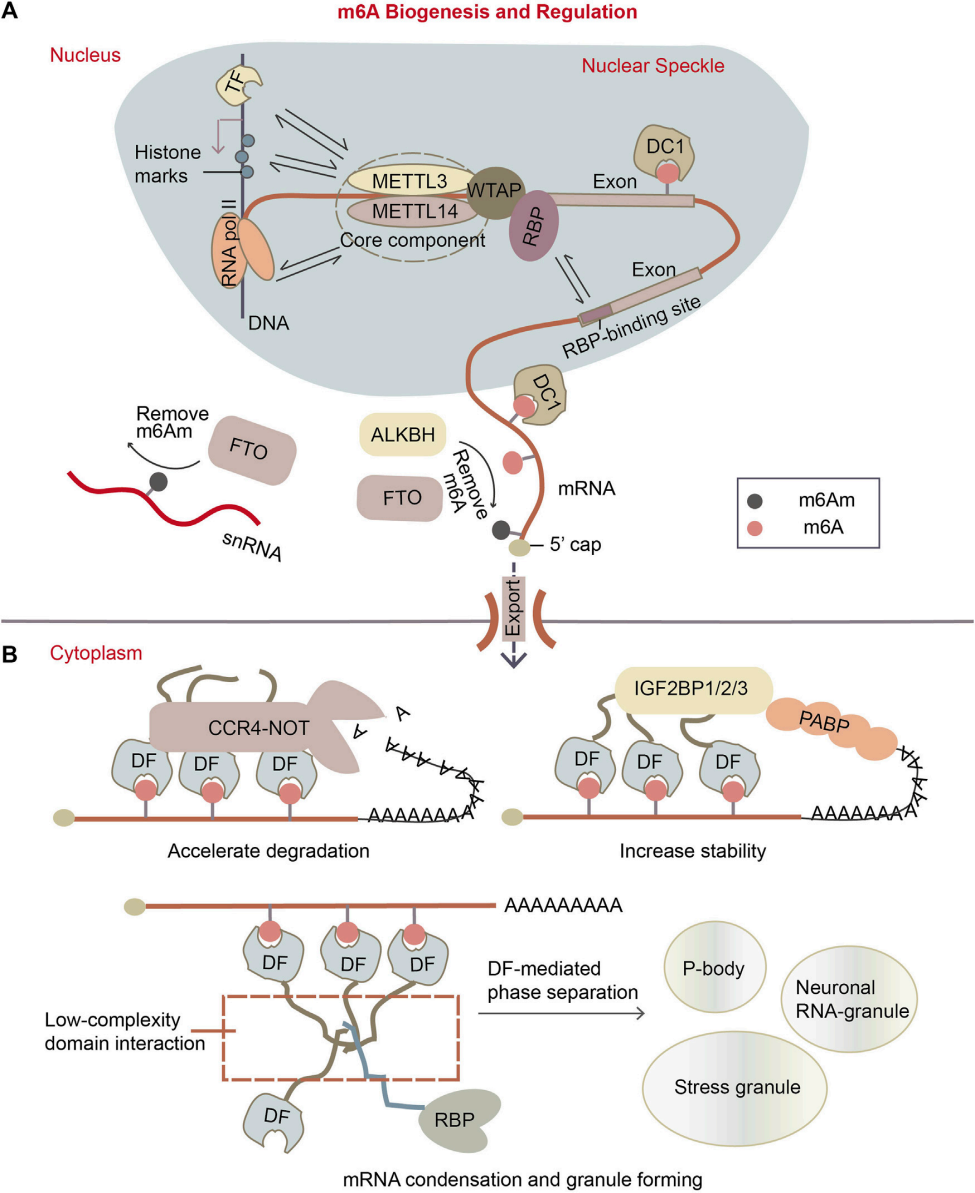
m^6^A biogenesis and regulation. **(A)** m^6^A is installed to mRNAs in the nucleus co-transcriptionally. The m^6^A writer complex, which comprises the core component of methyltransferase-like protein 3 (METTL3)/14 (METTL14) heterodimer and its accessory proteins. It is located in the nuclear speckle. It targets the potential m^6^A sites by recruiting RBPs as adapters, it can methylate A site in “DRACH” motif near the RBP-binding sites. Or, writer complex might be recruited to transcription loci by transcription factors (TFs) or histone marks, so methylation can also happen on some specific mRNAs. The m^6^A erasers are largely localized in the nucleus as well. The main m^6^A eraser acting on m^6^A on mRNA is ALKBH5. Fat mass and obesity associated protein (FTO) is found to preferentially target m^6^Am, especially on small nuclear RNAs (snRNAs). While in the nucleus, m^6^A can be recognized by specific nuclear reader proteins, mainly YTHDC1 (DC1), which may affect nuclear processes such as transcription, splicing, mRNA exportation. **(B)** Upon mRNA exports to the cytoplasm, m^6^A is recognized by specific reader proteins like YTHDF1/2/3 (DF1/2/3) that affect mRNA stability and localization of the mRNA. DFs mediate the degradation of m^6^A mRNAs by recruiting CCR4-NOT deadenylation complex, while the IGF2BP1/2/3 (insulin-like growth factor 2 mRNA-binding proteins) enhance m^6^A mRNA stability. Besides, DFs can make mRNA condensates through the low-complexity domain of proteins, forming granules like p-bodies, stress granules and neuronal RNA granules.

Extensive exploration has elucidated the major proteins involved in m^6^A pathway. These factors can be categorized as writers for m^6^A synthesis, erasers for m^6^A removal, and readers for m^6^A recognition. The m^6^A modification is deposited on mRNA co-transcriptionally in the nucleus (Ke et al., 2017) by a ∼1 MDa m^6^A writer complex. This complex consists of a hetero-dimer core component, methyltransferase-like 3 (METTL3) (Narayan and Rottman, 1988) and methyltransferase-like 14 (METTL14) (Figure 1A) (Liu et al., 2014). The crystal structure of METTL3-METTL14 complex showed that METTL3 is the catalytic subunit transferring a methyl group from donor SAM (S-adenosyl methionine) to acceptor adenine to form m^6^A (Wang C. et al., 2016; Wang P. et al., 2016; Sledz and Jinek, 2016). Knocking out METTL3 in *Arabidopsis* (Zhong et al., 2008), yeasts (Agarwala et al., 2012) or mammalian cells (Geula et al., 2015) results in complete or near-complete m^6^A depletion in poly(A)-tailed RNAs. Although METTL14 lacks SAM-binding domain and catalytic activity, it is known as an essential partner of METTL3, cooperating with METTL3 on their substrate RNA capture (Wang C. et al., 2016; Wang P. et al., 2016; Sledz and Jinek, 2016). In addition to the core component, alternative accessory proteins were found in the writer complex. These include Wilm’s tumor 1 associated protein (WTAP) (Schwartz et al., 2014b; Liu et al., 2014; Ping et al., 2014), Vir like m^6^A methyltransferase associated (VIRMA) (Horiuchi et al., 2013; Schwartz et al., 2014b), zinc finger CCCH-type containing 13 (ZC3H13) (Knuckles et al., 2018), HAKAI (Ruzicka et al., 2017; Bawankar et al., 2021; Wang et al., 2021) and RNA binding motif protein 15/15B (RBM15/15B) (Horiuchi et al., 2013; Patil et al., 2016). WTAP was reported to be an essential adaptor of METTL3 and METTL14, guiding their localization to the nuclear speckles, the loci of splicing and transcription (Ping et al., 2014). VIRMA interacts with WTAP, and depletion of VIRMA causes substantial loss of m^6^A (Haussmann et al., 2016; Lence et al., 2016; Kan et al., 2017). Specially, RBM15 mediates the binding of m^6^A methyltransferase complex to the U-rich RNA region adjacent to DRACH motif in a WTAP-dependent way (Patil et al., 2016). This model explained how m^6^A sites are selected from the highly-frequent consensus DRACH (D refers to G, A or U; R refers to G or A; H refers to A, C or U) sequences on mRNAs (Dominissini et al., 2012; Meyer et al., 2012; Fu et al., 2014; Linder et al., 2015) (Figure 1A). Other m^6^A writers such as METTL16 (Pendleton et al., 2017; Satterwhite and Mansfield, 2022) and a zinc finger protein ZCCHC4 (Ma et al., 2019) were identified to catalyze m^6^A synthesis on a subset of mRNAs, snRNAs, and rRNAs in the different sequence and structure context.

Two proteins FTO (fat mass and obesity-associated protein) and ALKBH5 (α-ketoglutarate-dependent dioxygenase alkB homolog 5) were reported to demethylate m^6^A on mRNA (Figure 1A). FTO protein was determined as demethylase of m^6^A on mRNA *in vitro* and *in vivo* (Jia et al., 2011; Fu et al., 2013). Later, it was revealed that FTO can also demethylate another similar modification m^6^Am (*N* ^6^, 2′-O-dimethyladenosine) on snRNA and cap-m^6^Am on mRNA (Mauer et al., 2017; Mauer et al., 2019). FTO protein is mainly localized in the nucleus, but it was also found in the cytoplasm in particular cell lines (Gulati et al., 2014). ALKBH5 was defined as another m^6^A eraser on mRNA (Zheng et al., 2013). It is primarily located in the nucleus (Linder et al., 2015) and is most highly expressed in testis. ALKBH5-mediated m^6^A downregulation influences several cellular pathways such as germ cell development and tumor cell proliferation (Zheng et al., 2013; Zhang et al., 2016a; Zhang et al., 2016b; Zhang et al., 2017).

The molecular function of m^6^A is majorly mediated by m^6^A readers with YTH domains, which are capable of recognizing and binding to m^6^A modification (Luo and Tong, 2014; Theler et al., 2014). These readers include nuclear protein YTHDC1 (DC1) and cytoplasmic proteins YTHDF1/2/3 (DF1/2/3). DC1 regulates mRNA transcription, splicing, and nuclear export through binding to m^6^A (Figure 1A) (Xiao et al., 2016; Roundtree et al., 2017b), while its paralog DC2 is unlikely involved in m^6^A pathway due to its low affinity to m^6^A (Li et al., 2022; Saito et al., 2022). There are still some arguments about what exactly DF proteins do on mRNA. Early research reported that DF paralogs exert different effects on their target mRNAs: DF1 and DF3 promote mRNA translation while DF2 accelerates mRNA degradation (Wang Y. et al., 2014; Wang et al., 2015; Shi et al., 2017). However, recent studies proposed that DF1/2/3 proteins likely have redundant functions without distinguishable preference for particular m^6^A sites (Lasman et al., 2020; Li et al., 2020; Zaccara and Jaffrey, 2020), and all three DF paralogs can recruit the deadenylation complex CCR4-NOT, thereby decreasing transcript stability (Figure 1B) (Zaccara and Jaffrey, 2020). DF factors can also be condensed through their low-complexity domains (Patil et al., 2018), forming functional phase-separated liquid droplets like p-bodies, stress granules or other RBP granules (Figure 1B) (Ries et al., 2019). Moreover, DF proteins have interaction with various RNA-binding proteins according to proximity labeling experiment (Zaccara and Jaffrey, 2020). This suggests that they possibly regulate mRNA metabolism in different ways through forming diverse reader complexes.

The m^6^A modification takes part in many biological and pathological processes so a variety of disorders occur once the m^6^A distribution, stoichiometry or readers changed in cells. It was reported that the m^6^A pathway regulates the balance between cell pluripotency and differentiation during organismal development (Batista et al., 2014; Chen et al., 2015). Moreover, altered m^6^A levels on specific gene transcripts are relevant to cancers. The decreased m^6^A levels on NANOG or FOXM1 mRNAs make the mRNAs stable, leading to the increase in cancer stem cells in breast cancer and glioblatoma (Zhang et al., 2016a; Zhang et al., 2017). On the contrary, the elevation of m^6^A level on oncogene c-myc mRNA enhances the stability and translation of the transcripts, and thus promotes the self-renewal and proliferation of the leukemia stem cells (Barbieri et al., 2017; Weng et al., 2018; Wang J. et al., 2020; Yankova et al., 2021). The m^6^A modification also accompanies viral infection (Lichinchi et al., 2016; Wu et al., 2019). HIV infection increases m^6^A levels on both virus and host mRNAs, and reducing m^6^A by either downregulation of writers METTL3/METTL14 or upregulation of eraser ALKBH5 suppresses HIV replication. All these observations revealed the functional significance of the m^6^A pathway.

There are still some arguments in the field regarding m^6^A biology, regulation, and function. The classic view believed that m^6^A is a reversible and dynamic modification, meaning that it can be methylated and demethylated in a regulated manner through its lifecycle (Roundtree et al., 2017a). Recent theory proposed that m^6^A modification is more likely static, determined by gene architecture, for example the lengths and distribution of exons and introns, and the main role of m^6^A in the cytoplasm is to mark mRNAs for degradation (Murakami and Jaffrey, 2022). The arguments are mainly focused on whether, how and when m^6^A positions and levels are regulated, and what are the main effects of m^6^A modification on mRNA. It is important to examine whether m^6^A regulation at installing stage is owing to recruitment of writer complex to certain chromatin loci by some transcription factors or epigenetic marks, and whether it is achieved through alterations in the composition or activity of writer complex. Additionally, it is interesting to investigate whether there are more writers, erasers, and readers that function in either the nucleus or the cytoplasm, enabling dynamically reversible m^6^A modifications. The development of sophisticated m^6^A profiling techniques, especially those can provide stoichiometry information for each identified m^6^A site, will help us know more about m^6^A biology, regulation, and function.

**FIGURE 2:**
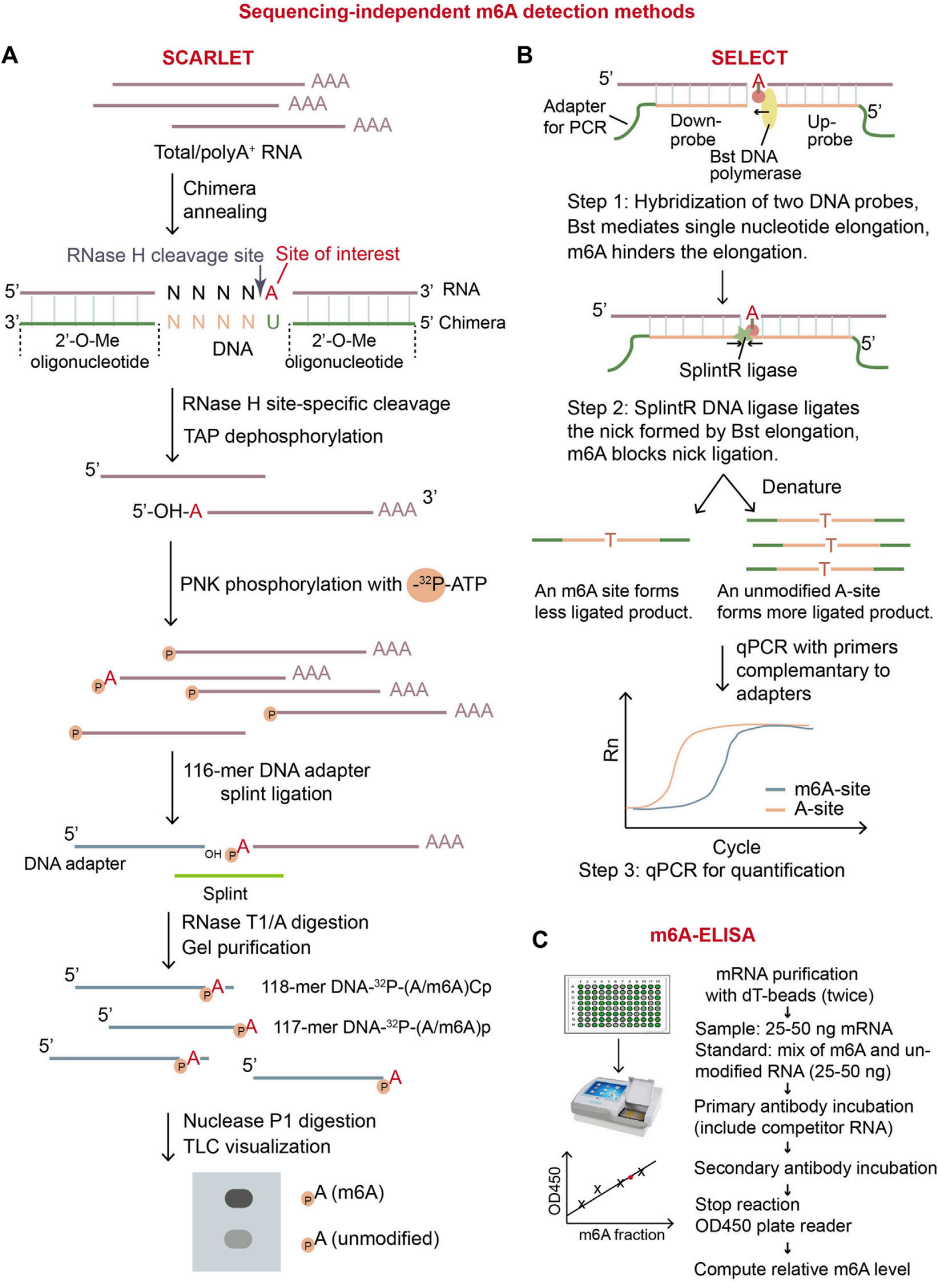
Overview of three sequencing-independent m^6^A detection methods. **(A)** Schematic diagram of SCARLET. In this method, specific site of interest is labeled with ^32^P, and then separate and visualized by TLC. The intensity of the signals of dots representing m^6^A ribonucleotide and A ribonucleotide will precisely quantify the fraction of m^6^A on the specific site. **(B)** Schematic diagram of SELECT (Xiao et al., 2018). This method exploits the principle that m^6^A modification disturbs DNA elongation and nick ligation at the modified position. Therefore, the quantity of the resulting ligated-DNA oligo represents modification status of A-site on RNA. **(C)** Illustration of m^6^A-ELISA. This method can be easily conducted by using commercial m^6^A-ELISA kit. The major steps are listed in the diagram. TAP: thermosensitive alkaline phosphatase; PNK: polynucleotide kinase.

In the past decade, many methods were developed to detect m^6^A locations and quantify m^6^A levels on mRNA. They can be categorized into sequencing-independent biochemical methods, SGS-dependent methods, and TGS-dependent methods. The sequencing-independent methods adopted digestion, qPCR, or ELISA to measure m^6^A (Figure 2). These methods can only quantify total m^6^A levels on mRNA or measure m^6^A at individual sites. As the next/second generation sequencing (NGS/SGS) and third generation sequencing (TGS) have undoubtedly become a major force driving the progression of life science (Goodwin et al., 2016), many high throughput-sequencing-based strategies, which aimed at transcriptomic m^6^A profiling, have been rapidly developed. Based on the ways to capture m^6^A sites, they are categorized as anti-m^6^A antibody-dependent and antibody-independent methods. The first-launched method meRIP-seq relied on anti-m^6^A antibody (Dominissini et al., 2012), which has led a trend in popping up antibody-dependent methods (Supplementary Table S1). Even though these methods have made a valuable contribution, they are limited majorly by the promiscuous nature of the antibody (Schwartz et al., 2013; Schwartz et al., 2014a; Linder et al., 2015; McIntyre et al., 2020) and the absence of high-resolution stoichiometry information. To overcome these problems, antibody-free methods have been developed (Supplementary Table S1). These methods were focused on distinguishing unmodified A-base from m^6^A, taking advantage of RNA enzymes such as m^6^A eraser FTO (m^6^A-SEAL-seq), RNA-editing enzyme APOBEC1 (DART-m^6^A-seq) or TadA (eTAM-seq), RNA endonuclease MazF (Mazter-seq) (Meyer, 2019; Pandey and Pillai, 2019; Zhang et al., 2019; Wang Y. et al., 2020; Hu et al., 2022; Xiao et al., 2023). In addition, some methods captured m^6^A sites by *in vitro* chemical labeling of nitrite-mediated deamination (GLORI) or metabolic labeling of allyl-modified SAM analogs (m^6^A-label-seq and m^6^A-SAC-seq) (Shu et al., 2020; Ge et al., 2023; Liu et al., 2023), while others employed algorithms to predict m^6^A directly with TGS data (Liu et al., 2019; Leger et al., 2021; Pratanwanich et al., 2021).

In this review, we will introduce current m^6^A detection methods employing sequencing-independent biochemistry or SGS/TGS technology, and discuss their strengths and limits.

## 2 Sequencing-independent m^6^A detection methods

Several sequencing-independent biochemical methods can detect and quantify m^6^A on RNA. These methods include the digestion-based LC/MS (liquid chromatography-tandem mass spectrometry) and SCARLET (Site-specific Cleavage and Radioactive Labeling followed by ligation-assisted Extraction and Thin-layer chromatography), the qPCR-based SELECT (single-base elongation-and ligation-based qPCR amplification method), and ELISA-based m^6^A-ELISA (Figure 2). Importantly, m^6^A-ELISA and LC/MS can quantify the m^6^A levels in total mRNA while SCARLET and SELECT can examine m^6^A status in individual sites of interest.

The m^6^A-ELISA method is commercially available. The m^6^A-ELISA kit provides a standard method to calculate the total m^6^A levels in RNA samples by using anti-m^6^A antibody to label m^6^A-containing RNA (Figure 2C) (Bringmann and Luhrmann, 1987; Ensinck et al., 2023). The advantages of m^6^A-ELISA are obvious. It is easy to conduct that the whole protocol can be finished in less than a day. It is cost effective and convenient as commercial ELISA kit is available. It has the potential to be adapted for detection of any modifications on RNAs. On the contrary, its disadvantages include limited sensitivity and the absence of location information of m^6^A. To summarize, m^6^A-ELISA can be applied to determine relative levels of total m^6^A in multiple samples.

LC/MS is a commonly-used, total m^6^A quantification method based on digestion (Thuring et al., 2017; Zaccara et al., 2019; Mathur et al., 2021). In this method, the phosphodiester bonds in purified mRNAs are hydrolyzed with nuclease P1, and the generated nucleoside 5′-monophosphates are further dephosphorylated with alkaline phosphatase for LC-MS analysis. It is an extremely accurate, sensitive, and quantitative method in which m^6^A nucleoside produces a characteristic mass spectrum (Thuring et al., 2017). The digestion-based LC/MS method can only quantify the total m^6^A level in mRNA, but not reveal modified gene identities. Also, the contamination from other abundant RNAs such as rRNA will affect the quantification accuracy.

Another widely-used method SCARLET aims to quantify m^6^A at a given site (Figure 2A) (Liu et al., 2013). In digestion-based method SCARLET, the chemically-modified oligonuleotide (2′-OMe)_6–8_(2′-H)_4_(2′-OMe)_6–8_ first hybridizes to the target m^6^A region on mRNA. Then, RNase H cleaves the hybrid mRNA site specifically at 5′ of the target adenosine regardless of modified or not. The 5′ end of the adenosine is further labeled with ^32^P and the radio-labelled mRNA fragment is splint-ligated with a 5′ adaptor, single-stranded 117-mer DNA, using DNA ligase. The RNA part in the ligated fragment is digested with RNase T1/A, the remaining adaptor DNA part with ^32^P-labeled unmodified-adenosine or m^6^A at its 3′end (117-mer DNA-^32^P-(A/m^6^A)p and 118-mer DNA-^32^P-(A/m^6^A)Cp) is gel-purified, digested into mono-nucleotides using nuclease P1. The ^32^P-labeled adenosine or m^6^A is visualized and quantified by thin-layer chromatography (TLC) (Liu et al., 2013). Although SCARLET is laborious and requires to use radioactive isotope, it can accurately quantify m^6^A level in a specific site at single-base resolution. Therefore, it has been used as the “gold standard” in m^6^A stoichiometry for individual desired sites (Zaccara et al., 2019).

An economic and time-saving qPCR-based method, SELECT, can also determine m^6^A status at individual sites (Figure 2B) (Xiao et al., 2018). SELECT exploits the ability of m^6^A to hinder both the single-base elongation by DNA polymerase Bst and the nick-ligation by ligase SplintR (Xiao et al., 2018). In SELECT, two DNA probes, including adapters for qPCR, hybridize to the mRNA region flanking an adenosine site (A-site) of interest. After treatment of Bst and SplintR, if the A-site of interest is unmodified, two DNA probes are ligated efficiently, forming a large number of the full DNA fragments with both up and down adapters; otherwise, if the A-site is modified, two probes are less likely to be ligated, forming very few of the full DNA fragments with both adapters. The full DNA fragments are later amplified by qPCR for quantification. Only the ligated DNA with both up and down adapters can be amplified in qPCR, thus, the value of threshold cycle (CT) of qPCR reflects the initial m^6^A stoichiometry of the given site. In the experiment, another unmodified A-site near the m^6^A site of interest on the same mRNA should be used for quantification of the input mRNA with the given m^6^A site. SELECT is not able to discriminate different modification types on adenosine, meaning that m^6^A, m^1^A, and Am will give similar decrease in qPCR signals compared to unmodified adenosine. To validate the modification type, SELECT requires additional RNA samples which have undergone *in vitro* treatment with m^6^A eraser or *in vivo* depletion of m^6^A biogenesis factors. In general, SELECT can achieve absolute quantitation by introducing standard curves and has potential for wider use in the future because of its flexibility and convenience.

These biochemical methods can quantify the m^6^A levels in total RNA or at individual sites, however, they cannot give us a comprehensive view on m^6^A distribution and stoichiometry at a transcriptome level. So, many strategies based on high-throughput sequencing have been emerged.

## 3 SGS-based m^6^A detection methods

### 3.1 Methods relying on anti-m^6^A antibody

#### 3.1.1 MeRIP-seq

**FIGURE 3:**
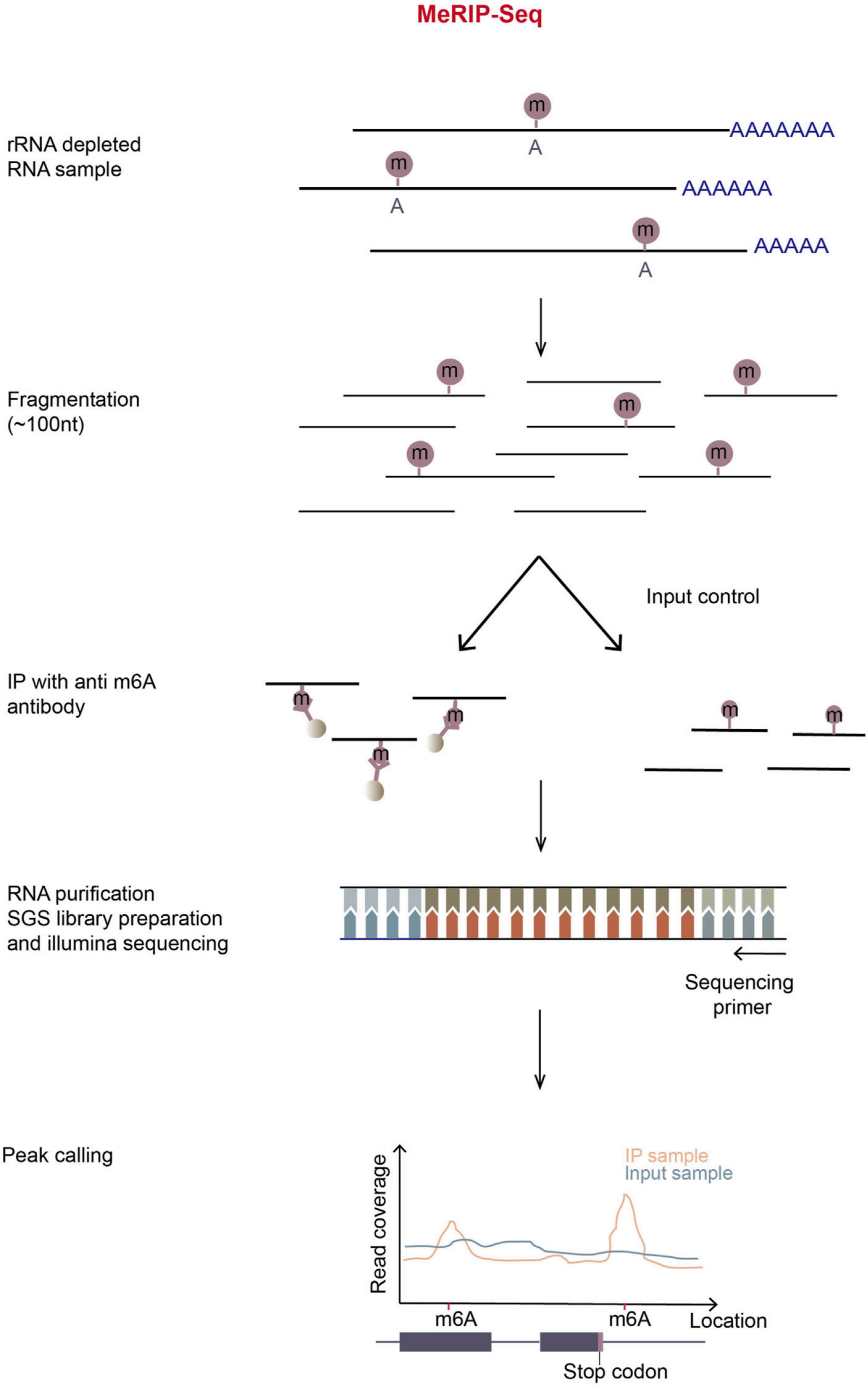
Schematic representation of MeRIP-Seq. In MeRIP-seq, rRNA-depleted or polyA-enriched RNA samples are obtained by treating total RNA with the RiboMinus kit or oligo-dT-conjugated beads and then chemically fragmented with metal ions in appropriate incubation temperature and time. A portion of fragmented RNA is saved for input control, and the residual/remaining RNA is immunoprecipitated using m^6^A-antibody-coupled Dynabeads. Immunoprecipitation (IP) can be repeated to enhance signal-noise ratio. The input and m^6^A-enriched RNA fragments are then subjected to the normal RNA-seq library preparation process. The libraries are then sequenced in illumina sequencing platform. The subsequent reads generated are aligned to the reference genome, most reads will pile up at m^6^A locations in the IP sample. By calculating the read coverage at each location, peaks can be observed around the m^6^A locations in MeRIP samples.

MeRIP-Seq (or m^6^A-seq) is the first-developed and most-widely-used method for transcriptomic profiling m^6^A sites on mRNA. In this method, mRNA is randomly fragmented and immunoprecipitated using m^6^A-specific antibody. Next, RNA-seq libraries are prepared from input and immunoprecipitated RNA according to standard protocol (Figure 3). In theory, the sites closer to m^6^A will have the higher read coverage. Therefore, when the meRIP sequencing reads are mapped to a reference genome, m^6^A sites will be centered in the read peaks (Dominissini et al., 2012; Meyer et al., 2012).

MeRIP-Seq is relatively stable, convenient, fast, and cost-effective, and can be used in large scale experiments (Zhang et al., 2022). In spite of its usefulness, this method requires a large amount of starting RNA and has high noise background. Furthermore, anti-m^6^A antibodies possibly mis-recognize other modifications similar to m^6^A, such as m^6^Am. Also, only the regions with high methylation levels can be identified with low resolution of m^6^A sites.

To overcome the low resolution and high background problems, UV-crosslinking-based m^6^A mapping methods giving nucleotide resolution were subsequently developed (Linder et al., 2015; Koh et al., 2019; Roberts et al., 2021).

#### 3.1.2 miCLIP, meCLIP and m^6^ACE-seq

**FIGURE 4:**
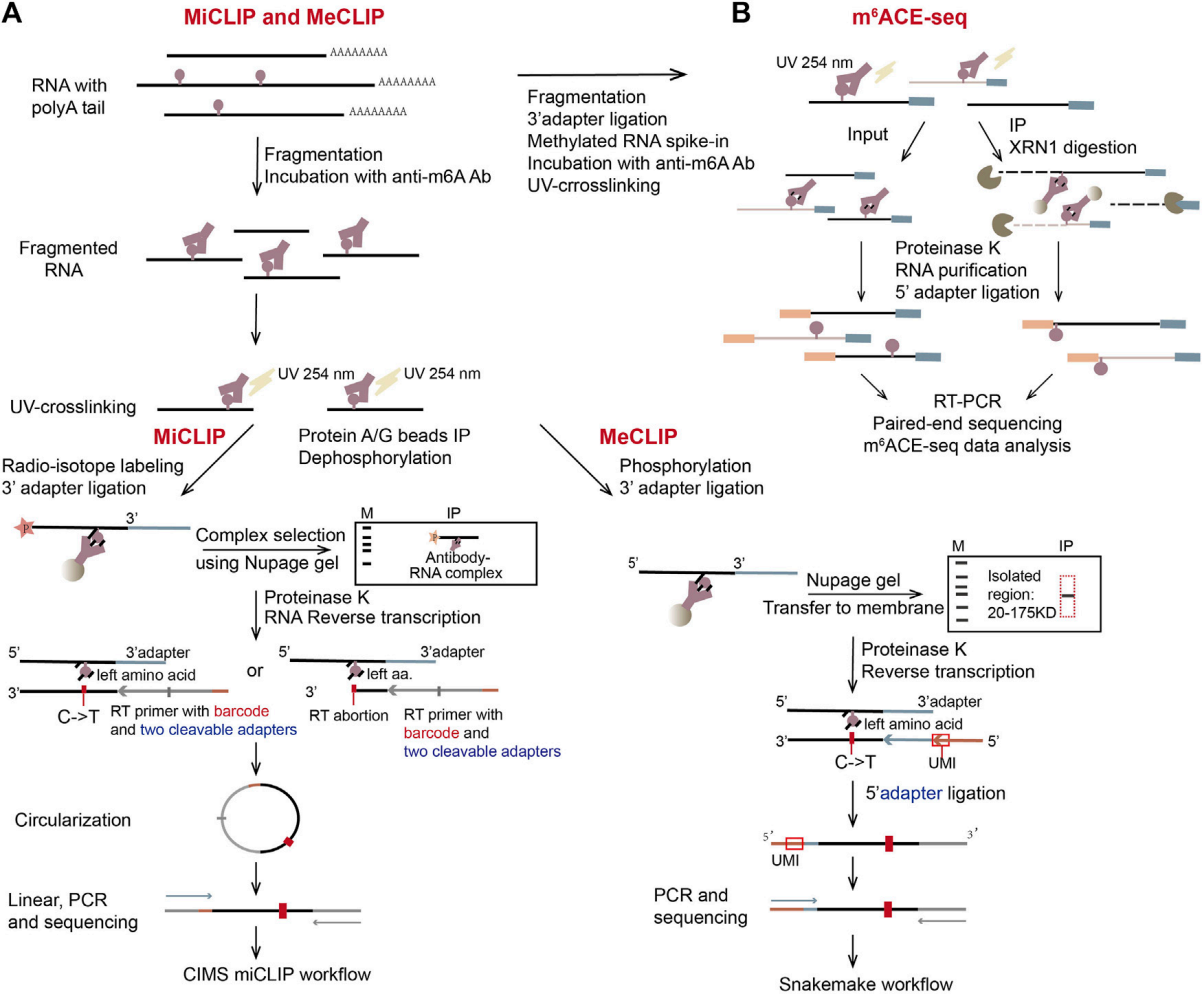
Schematic representation of three CLIP-based methods, miCLIP, meCLIP, and m^6^ACE-seq. **(A)** The representation of miCLIP and meCLIP. The two workflows are similar. Three main differences are: fragmented RNAs (100 nt–200 nt) are longer in meCLIP than those in miCLIP; meCLIP omits the radio-labeling step and its procedure includes size-specific isolation of the RNA-antibody complex with the protein ladder in NuPAGE gel; meCLIP ligates 3′ adaptor and 5′ adaptor separately instead of the cDNA circularization. **(B)** The representation of m^6^ACE-seq. Instead of gel purification, m^6^ACE-seq utilizes 5′ to 3′ exonuclease XRN1 to digest the antibody-crosslinked mRNAs from their 5′ ends.

miCLIP (m^6^A individual-nucleotide-resolution cross-linking and immunoprecipitation) has a similar idea to iCLIP method which clarifies the protein-binding sites on RNA (Figure 4A, left) (Konig et al., 2010; Linder et al., 2015). In miCLIP, anti-m^6^A antibody and fragmented mRNA are incubated together, then the antibody and m^6^A on mRNA are UV-crosslinked. The RNA-antibody complex is immunoprecipitated and the 5′ end of RNA is radioactively labeled using [γ-^32^P] ATP through PNK (polynucleotide kinase) reaction. Next, through the denaturing NuPAGE gel-run and the nitrocellulose membrane-transfer, the antibody-RNA complex is separated, visualized and purified. Partial digestion with proteinase K leaves peptide residues on m^6^A nucleotides, which leads to abortion or introducing C-to-T nucleotide transition at m^6^A positions during reverse transcription (RT) (Linder et al., 2015). miCLIP can therefore achieve nucleotide resolution in determining m^6^A sites. However, the radio-isotope labeling will complicate the experiment and low UV-crosslinking efficiency will decrease library complexity. Another CLIP-based technique, MeCLIP (m^6^A eCLIP) (Roberts et al., 2021) has simplified the process by omitting the steps of radio-labeling and visualizing RNA (Figure 4A, right). In addition, two linear adapters are ligated to RNA/cDNA fragments separately and unique molecular identifier (UMI) is included in the RT primer, which has improved library complexity of MeCLIP.

In 2019, another CLIP-based method m^6^ACE-seq (m^6^A-cross-linking-exonuclease sequencing) was developed (Koh et al., 2019). In this method, m^6^A and its antibody are UV-crosslinked and immunoprecipitated, then RNA-antibody complex is digested with a 5′-to-3′ exonuclease XRN1 (Figure 4B). The RNA digestion will stop at crosslinked m^6^A positions, by which m^6^A sites are precisely located at single nucleotide resolution (Koh et al., 2019). Compared to the other two CLIP-based methods, m^6^ACE-seq has abandoned most of the complicated steps. Instead, it uses XRN1 to digest mRNA fragments from the 5′ ends to the first m^6^A positions, which are protected by crosslinked antibody. Therefore, the following NGS paired-end RNA-seq can produce reads that pile up at locations of m^6^A, achieving single nucleotide resolution in m^6^A detection.

### 3.2 Methods relying on enzymes

#### 3.2.1 DART-m^6^A-seq

Antibody-free methods have been developed since 2019. The first one is DART-seq (deamination adjacent to RNA modification targets sequencing) (Meyer, 2019; Flamand and Meyer, 2022). The method employs a RNA modification enzyme-based fusion protein system. The fusion protein methodology has been widely applied to detect interactions between RNA-binding proteins (RBPs) and their target RNAs by constructing the fusion protein of a given RBP with an RNA-modification enzyme (Lapointe et al., 2015; McMahon et al., 2016; Jin et al., 2020; Brannan et al., 2021; Piao et al., 2023). It has also been applied for CRISPR-Cas9-based genome editing, where deactivated Cas9 (dCas9) is fused with the nucleic-acid-editing enzyme APOBEC1 to edit desired bases in target genomes under the guidance of guide RNAs (gRNAs) (Komor et al., 2016).

**FIGURE 5:**
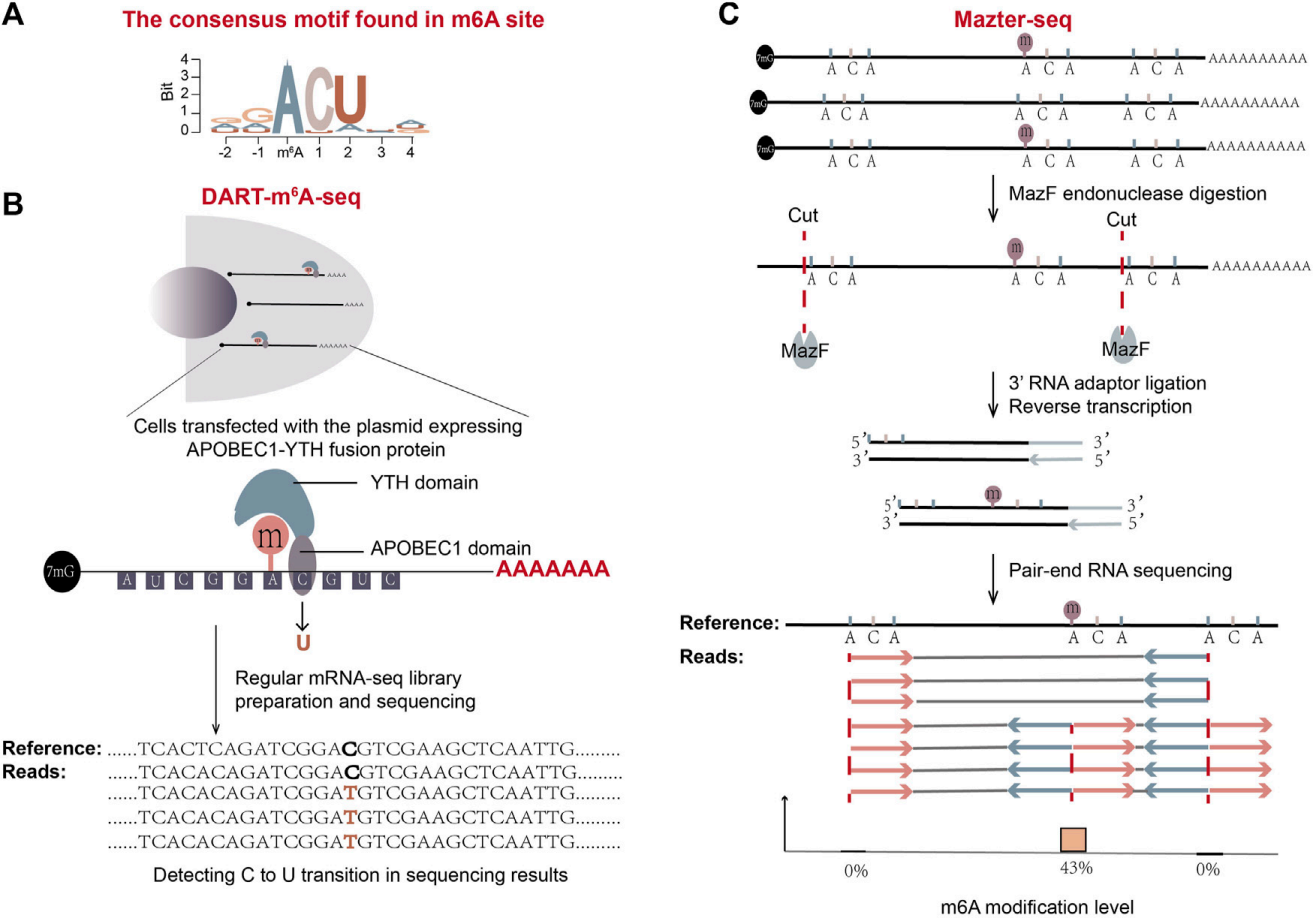
Schematic representation of two antibody-free methods: DART-m^6^A-Seq and Mazter-Seq. **(A)** The consensus motif found in m^6^A sites is shown. **(B)** The workflow of DART-m^6^A-seq. **(C)** The workflow of Mazter-seq (Garcia-Campos et al., 2019). After digestion with MazF, the generated RNA fragments mostly start with 5′ACA. The following standard pair-end RNA-seq gives sequencing reads that are of internal or terminal ACA. The ratio of sequencing reads with internal ACA to reads split at 5′ ends of ACA represents the relative quantity of methylation.

DART-seq takes advantage of the fusion protein of an RNA-editing enzyme APOBEC1 and the YTH domain of m^6^A reader protein YTHDF2 to mark m^6^A sites both in cells and *in vitro* (Figure 5B) (Meyer, 2019). When the fusion protein is expressed in cells, the YTH domain in the fusion protein (Wang X. et al., 2014; Schwartz et al., 2014b) is able to be recruited to the m^6^A sites. Its APOBEC1 domain could then deaminate adjacent cytosines to uracils in cells. These C-to-U conversions can be identified by conventional mRNA-seq in short-read SGS or long-read TGS platform. Consistent with the known m^6^A-consensus motif DRm^6^ACH (Figure 5A), C-to-U transitions are frequently detected at cytosines adjacent to the motif.

DART-seq requires only a small amount of starting material and has a relatively high recurrence rate. In addition, when DART-seq is combined with a TGS long-read platform, it is able to profile mRNA isoform-specific methylation patterns (Meyer, 2019). Utilizing single-cell sequencing like 10x genomics SGS platform, DART-seq has achieved the single-cell level scDART-seq in 2022, and discovered instinct m^6^A signatures of individual cells independent from gene expression profiles (Tegowski et al., 2022). At the same time, this method has a short library construction time and simple operation. However, it is hardly employed in some primary cells with low transfection efficiency and is also time-consuming to clone the fusion protein-expressing plasmids. DART-seq accompanies overexpression artifact especially if protein expression time is long, thus an inducible promoter will be a good choice to express the fusion protein.

#### 3.2.2 Mazter-Seq

Another antibody-free m^6^A detection method, Mazter-Seq or REF-seq (m^6^A-sensitive RNA-Endoribonuclease-Facilitated sequencing, a similar method developed by another group) (Garcia-Campos et al., 2019; Pandey and Pillai, 2019; Zhang et al., 2019), utilizes a special type of bacteria-derived single-stranded RNA endoribonuclease MazF, whose activity is sensitive to the methylation status of RNA (Imanishi et al., 2017). MazF can recognize and cleave before the ACA motif. But in the case of m^6^A-CA, in which the first adenosine is methylated, it can’t (Figure 5C). When the extracted mRNA is digested with endoribonuclease MazF, unmethylated ACA sites generate two types of RNA fragments, one with 5′ terminal ACA sequence and the other with a 3′ terminal ending immediately before ACA. On the contrary, methylated m^6^A-CA sites generate RNA fragments with internal ACA sequence. The terminal features of sliced RNA fragments are then detected by NGS sequencing (Figure 5C). After alignment of reads to a reference genome, the number of reads split immediate-upstream of ACA and the number of reads spanning the ACA positions are counted for each ACA position. The proportion of reads with internal ACA represents the m^6^A level at the location (Garcia-Campos et al., 2019; Pandey and Pillai, 2019; Zhang et al., 2019).

The Mazter-Seq method has a low false positive rate and is less laborious than the antibody-based methods. Though it is able to provide stoichiometry information for each detected m^6^A site, there are two major drawbacks of this method. Firstly, the enzyme activity can be influenced by factors other than methylation state. For example, the secondary structure of RNA at an ACA site may decrease the cleaving efficiency of MazF at the site. Therefore, a parallel control experiment from a methyltransferase-knockout mutant is required to neutralize the background, which will add the complexity to the method. Secondly, because MazF enzyme is only able to slice an “ACA” motif among DRACH motifs, Mazter-Seq can determine m^6^A at ACA positions but not all possible positions. Data showed that its estimated detection rate of m^6^A sites is only about 20%.

#### 3.2.3 m^6^A-SEAL-seq

**FIGURE 6:**
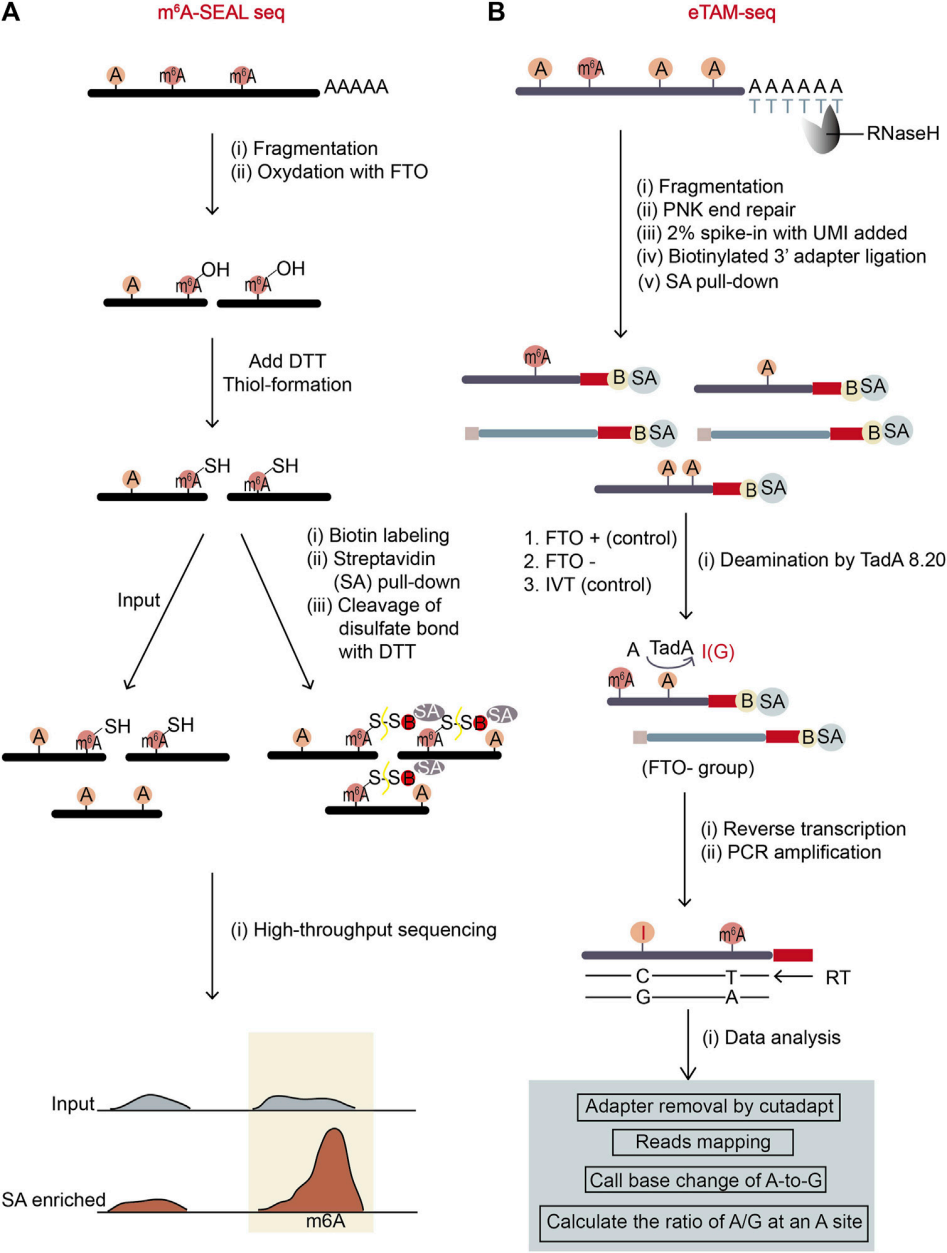
Schematic representation of two antibody-free methods, m^6^A-SEAL-seq and eTAM-seq. **(A)** The workflow of m^6^A-SEAL-seq. The sequential treatment of FTO enzyme and DTT makes m^6^A sites are biotinylated and enriched by streptavidin (SA)-biotin interaction. The m^6^A sites are represented by sharp peaks in m^6^A-pulldown samples. **(B)** The workflow of eTAM-seq. Unmodified adenosine in fragmented mRNA is turned into inosine by deaminase TadA8.20. Inosine will be recognized as G during NGS sequencing. Therefore, the precise locations and levels of m^6^A sites can be identified through finding A-sites with low percentages of A-to-G transitions.

Compared with Mazter-Seq, m^6^A-SEAL-Seq (Antibody-free, FTO-assisted chemical labeling method) (Wang Y. et al., 2020) has overcome the sequence context bias in m^6^A detection. It first uses FTO enzyme to oxidize m^6^A sites on the fragmented mRNAs *in vitro*. After m^6^A is transformed to unstable intermediate hm^6^A (*N* ^6^-hydroxylmethyladenosine), the added DTT further converts hm^6^A to more stable dm^6^A (*N* ^6^-dithiolsitolmethyladenosine) (Figure 6A). Next, the RNA fragments are divided into input and dm^6^A-pulldown groups, and the dm^6^A-pulldown group undergoes additional treatment. In dm^6^A-pulldown group, the free sulfhydryl group on dm^6^A reacts with MTSEA (methanethiosulfonate) on MTSEA-labeled biotin, a commercial thiol-reactive reagent. Later, the biotinylated dm^6^A is enriched with streptavidin (SA) beads, and the RNA fragments with dm^6^A are recovered by cleaving the disulfate bonds with DTT. The RNA fragments from input and dm^6^A-pulldown groups then undergo RNA sequencing respectively. The rough m^6^A positions will be revealed by the enriched sequencing peaks from the dm^6^A-pulldown group relative to the input group.

This method has complicated experimental procedure as its experimental environment should be controlled properly to retain the intactness of mRNA fragments. It also has low resolution and no stoichiometry information for each m^6^A site. True m^6^A sites may be omitted because of the limits on FTO catalytic capability and thiol-labeling efficiency. However, compared to the widely-used meRIP-seq, it is both time saving (the FTO incubation time is as short as 5 min) and cost saving (the usage of biotin-SA for enrichment costs less than the usage of antibody). Further, according to the original publication, the sites found in m^6^A-SEAL-seq are quite reliable as they overlapped with the sites from other methods to a certain degree.

#### 3.2.4 eTAM-seq

eTAM-seq (evolved TadA-assisted *N* ^6^-methyladenosine sequencing) is a new method hiring a deaminase to convert unmodified adenosine (A) to inosine (I) in RNA (Xiao et al., 2023). Since inosine reads as guanosine in sequencing, deaminated sites can be identified by A-to-G nucleotide alterations. TadA8.20 was engineered from an *E.coli* deaminase TadA, possessing robust deamination activity and minimal sequence context dependence (Figure 6B). This hyper-active enzyme can completely deaminate most of unmodified A but not m^6^A in RNA without breaking the RNA. Therefore, after treating purified RNA with TadA8.20, nearly 99% of A-bases at an unmodified A-site are deanimated and read as G. However, at an m^6^A site with a certain percentage of m^6^A modifiaction, unmodified A-bases read as G while m^6^A-bases read as A. So, the percentage of A-reads at an m^6^A site represents m^6^A level at the position. To ensure the accuracy and reduce false positive rates caused by the incomplete deamination of TadA8.20 enzyme, eTAM-seq sets two control groups: one is an RNA sample treated with the m^6^A eraser FTO, which can remove methyl-group from m^6^A *in vitro* (serving as a control to eliminate the bias from other A modifications which are also resistant to TadA8.20); the other is an *in vitro* transcription (IVT) sample which is an in-vitro-transcribed modification-free cell transcriptome mimic (serving as a control to eliminate the bias from the TadA8.20-not-accessible A-bases in transcriptome) (Figure 6B).

eTAM-seq has lots of merits. It has no sequence bias and provides stoichiometry information for detected m^6^A sites at single-nucleotide resolution. Importantly, it can quantitatively detect m^6^A with as few as ten cells in a simple workflow. However, the expression and purification quality of TadA8.20 may affect the sequencing result. To sum up, despite potential limitations, eTAM-seq is an advanced and practical method for m^6^A site detection and m^6^A level quantification.

### 3.3 Methods relying on SAM analogs

#### 3.3.1 m^6^A-SAC-seq

**FIGURE 7:**
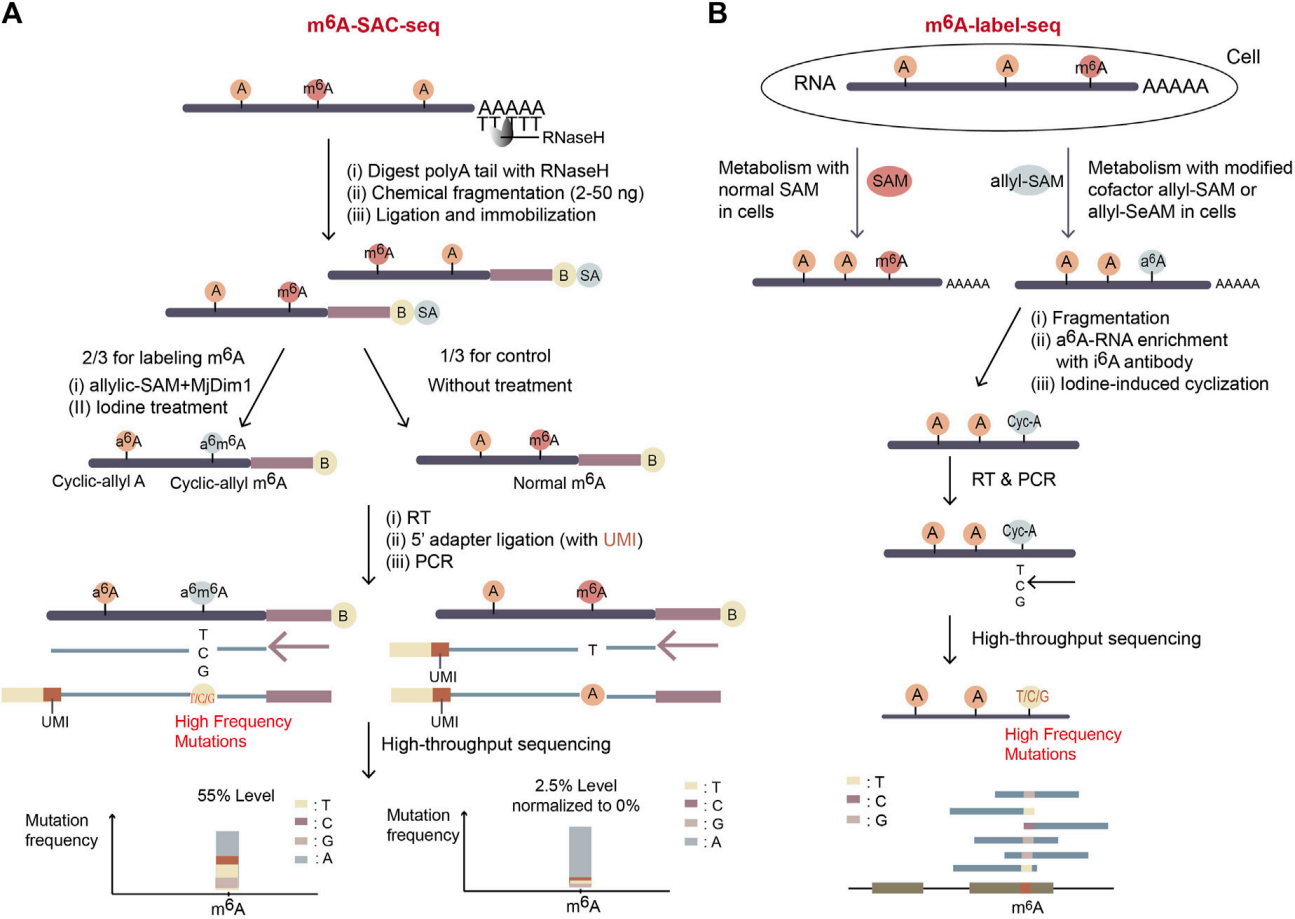
Schematic representation of two SAM analog dependent methods. **(A)** The workflow of m^6^A-SAC-seq. It depends on the dimethyltransferase MjDim1 and the cofactor allylic-SAM to turn m^6^A to a^6^m^6^A, which is further cyclized after iodine treatment. And therefore, original m^6^A sites gain high frequency of mutations during reverse transcription. Further, the mutation frequency at a potential m^6^A site indicates the m^6^A level of the site. If a site has mutation frequency more than 5% in the treated group and less than 2.5% in the control untreated group, the site will be considered as a potential m^6^A site. **(B)** The workflow of m^6^A-label-seq. The expected m^6^A sites on nascent RNA are metabolically labelled as a^6^A when exposing cells to allyl-SAM or allyl-SeAM. Further iodine-mediated cyclization of a^6^A sites dramatically increases mutation rates of RT at the sites. The m^6^A positions and levels are identified by NGS with extraordinary high mutation rates.

The method of m^6^A-SAC-Seq (m^6^A-selective allyl chemical labeling and sequencing) (Hu et al., 2022; Ge et al., 2023) takes advantage of MjDim1, a dimethyltransferase from *M. jannaschii*, which can methylate both unmodified adenosine and m^6^A into a dimethylated form of m^6,6^A (Figure 7A). The fragmented mRNA was treated with MjDim1 in the presence of a cofactor allylic-SAM instead of SAM. Under this condition, MjDim1 exhibits nearly tenfold preference for m^6^A over unmodified A-base, and catalyzes m^6^A into allyl-modified m^6^A (a^6^m^6^A) and unmodified A-base into allyl-modified A (a^6^A). Subsequent iodine (I2) treatment cyclizes these two substrates respectively, and the cyclized a^6^m^6^A (original m^6^A) generates a tenfold higher mutation rate than cyclized a^6^A (original unmodified A) in the following reverse transcription with HIV-1 reverse transcriptase (Figure 7A). Thus, the original m^6^A sites can be identified through their high mutation frequencies in mRNA-sequencing.

So far, since MjDim1 is biased to catalyse mRNA substrates with a more common GAC motif over a less frequent AAC motif, it is uncertain whether all m^6^A sites could be labeled equally in m^6^A-SAC-Seq. Further, the complex and time-consuming experimental procedure makes it difficult to use. Nevertheless, this method provides a promising way for m^6^A profiling since it is free from antibody consumption and rRNA-depleted input can be as low as 30 ng. Also, it reaches single-nucleotide resolution for measuring dynamic change in m^6^A distribution and stoichiometry of each site. m^6^A-SAC-Seq has an exciting prospect and can be applicable to many biological studies.

#### 3.3.2 m^6^A-label-seq

m^6^A-label-seq (metabolic labeling method detects m^6^A) (Shu et al., 2020), another method relying on the allyl-modified SAM analogs, was developed in 2020. To distinguish m^6^A from unmodified A-base, cells are incubated with allyl-SAM or allyl-SeAM so the expected m^6^A positions are metabolically labeled as *N* ^6^-allyladenosine (a^6^A). Fragmented a^6^A-containing RNA is enriched with the commercial *N* ^*6*^-isopentenyladenosine (i^6^A) antibody and a^6^A is converted to *N* ^*1*^, *N* ^*6*^-cyclized adenosine (cyc-A) by iodine treatment. Introduced mutations at cyc-A positions during reverse transcription indicate the m^6^A sites (Figure 7B).

m^6^A-label-seq can profile transcriptome-wide m^6^A positions at base resolution without sequence bias. However, the exposure of living cells to SAM analogs raises some concerns about altered biological pathways, for example, it was reported a weak alteration in gene expression related to endoplasmic reticulum stress and apoptotic signaling (Shu et al., 2020). Also, the step of a^6^A-RNA enrichment with i^6^A antibody possibly affects the m^6^A stoichiometry. Improvement of m^6^A-label-seq should focus on increasing m^6^A-labeling yield and meanwhile decreasing artificial effects.

### 3.4 Methods relying on chemicals—GLORI

**FIGURE 8:**
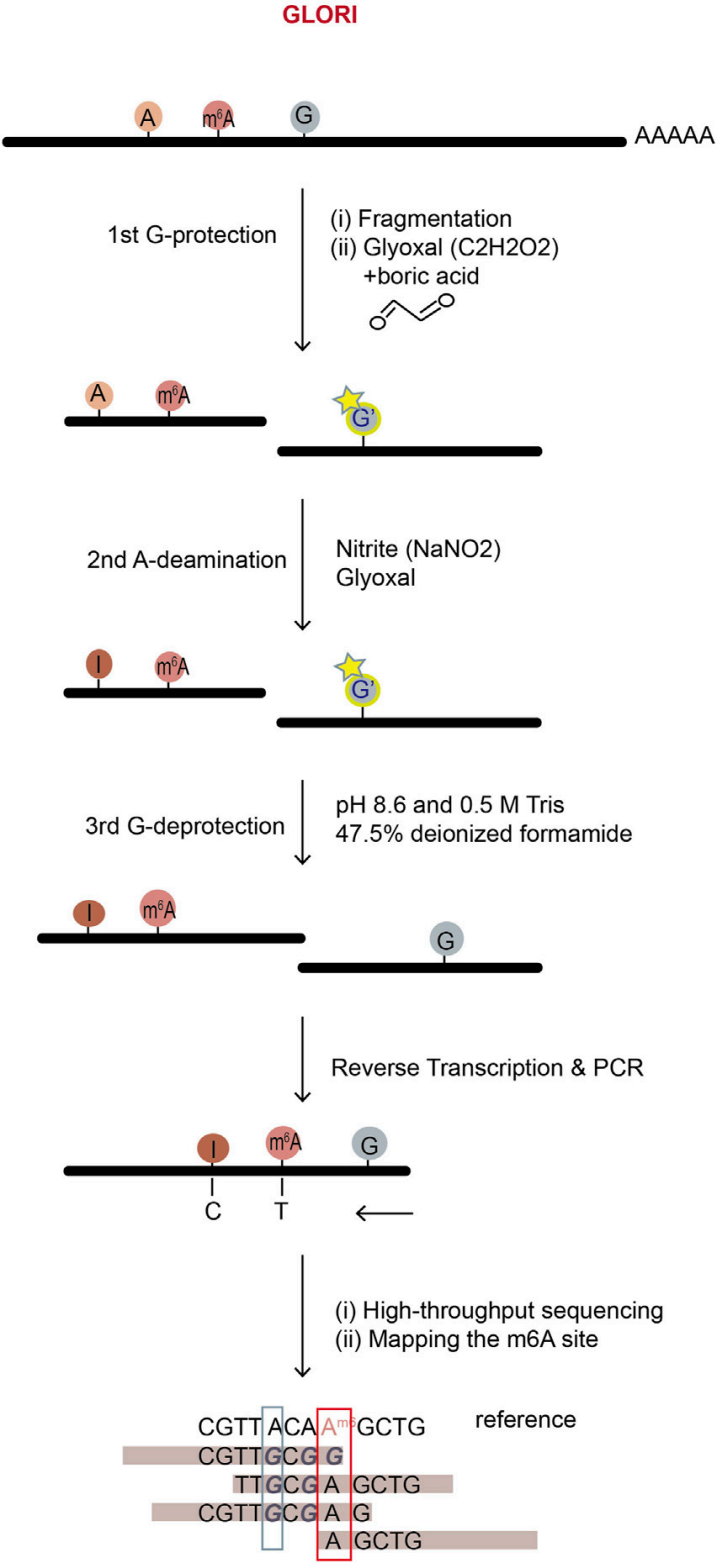
The workflow of GLORI m^6^A sequencing. GLORI uses nitrite to deaminate unmodified A into I (G), while m^6^A and G, C nucleotides are protected. Thus, nearly complete A-to-G transition represents an unmodified A-site; incomplete A-to-G transition represents an m^6^A site and the m^6^A level can be calculated by the ratio of A to (A + G) at the site in mRNA-sequencing.

GLORI (glyoxal and nitrite-mediated deamination of unmethylated adenosines), a new method developed in 2022, specifically deaminates unmethylated adenosine but not m^6^A using chemicals, glyoxal (C2H2O2) and nitrite (NaNO2), to locate the original m^6^A sites in RNA (Liu et al., 2023). It adopted a strategy similar to the bisulfite sequencing method for DNA 5 mC detection (Raiber et al., 2017). In GLORI, glyoxal is first used as a reagent to protect regular G-bases by reacting with their exocyclic amino groups, so the regular G-bases will not be deaminated to X during nitrosation step (Figure 8). Further, glyoxal works as a catalyst to accelerate the deamination of unmodified adenosines in the next step of nitrite existence condition, speeding up the desired A-to-I (G) reaction while concurrently reducing the side reaction of C-to-U deamination. In GLORI, A-to-G transition rate achieved ∼98.0%, with <1.0% G-to-X and ∼3.3% C-to-U transitions. After adenosine deamination, the glyoxal-protected G-bases are recovered to the regular G-bases under alkaline or heated condition. Then, because unmodified A was deaminated to I (G) but m^6^A was not, the following high-throughput sequencing reveals the m^6^A positions which didn’t undergo deamination (Figure 8).

GLORI fully depends on chemical reactions, so compared to the methods relying on enzymes, it works more stably with better repeatability and lower cost. It provides quite precise estimation of both m^6^A positions and stoichiometry using only 100 ng of mRNA. However, the extracted RNA has to undergo a series of complex chemical processing *in vitro*, which challenges the RNA integrity and might raise concern about mRNA degradation. Nevertheless, a simple and fast protocol at low cost makes GLORI have the potential to be widely used in the field.

## 4 TGS-based m^6^A detection methods

The advantages of TGS include simple sequencing library preparation and extremely long sequencing reads, making them suitable for RNA isoform measurement and long genome assembly. Even though TGS has relatively high error rates, development of TGS has pushed the advancement of m^6^A detection strategies (Saletore et al., 2012). Direct RNA sequencing in Oxford Nanopore Technology (ONT) platform and single-molecule, real-time (SMRT) reverse transcription in PacBio platform have been employed in m^6^A detection (Schadt et al., 2010; Vilfan et al., 2013; van Dijk et al., 2018; Abebe et al., 2022).

### 4.1 m^6^A detection using ONT direct RNA sequencing

Since the modified and unmodified ribonucleotides display differentiated signal patterns in Oxford direct RNA sequencing (DRS), through decoding these signals, DRS has provided a fascinating system for detection of RNA modifications. Thus, DRS combined with computational analysis is able to identify modified sites at single-nucleotide resolution and has been widely applied in profiling various RNA modifications, including those in RNA viruses (Liu et al., 2019; Kim et al., 2020; Leger et al., 2021; Pratanwanich et al., 2021; Abebe et al., 2022; Hong et al., 2022). In Oxford DRS, each consecutive 5-mer nucleotide inside the nanopore determines a blockage effect on the ionic current, so the patterns of current intensity change can be used to identify the transiting nucleotides regardless of whether modifications are present. Although extracting RNA modification information from DRS reads is still challenging, the issue is highly in need to solve (Keller et al., 2018; Zhang R. et al., 2021). New approaches have adopted the analysis of base call error rates or raw signals “squiggles” or both (Loose et al., 2016; Abebe et al., 2022).

#### 4.1.1 The algorithms based on base call error rates

In nanopore DRS, the raw current intensities are recorded in real time from an RNA molecule, forming a squiggle graph. Algorithms such as guppy are able to carry out base calls from raw signals, generating RNA sequences and also assigning a probability score for each nucleotide to signify the accuracy of the call (Soneson et al., 2019; Wick et al., 2019). Because m^6^A-modified nucleotides affect the ionic current differently from their unmodified counterparts, error rates are significantly high near the modified nucleotides. Therefore, algorithms, such as EpiNano, DRUMMER, Eligos2, JACUSA2, DiffErr, are able to identify m^6^A sites based on error rates of base calls (Parker et al., 2020; Price et al., 2020; Jenjaroenpun et al., 2021; Liu et al., 2021; Piechotta et al., 2022).

**FIGURE 9:**
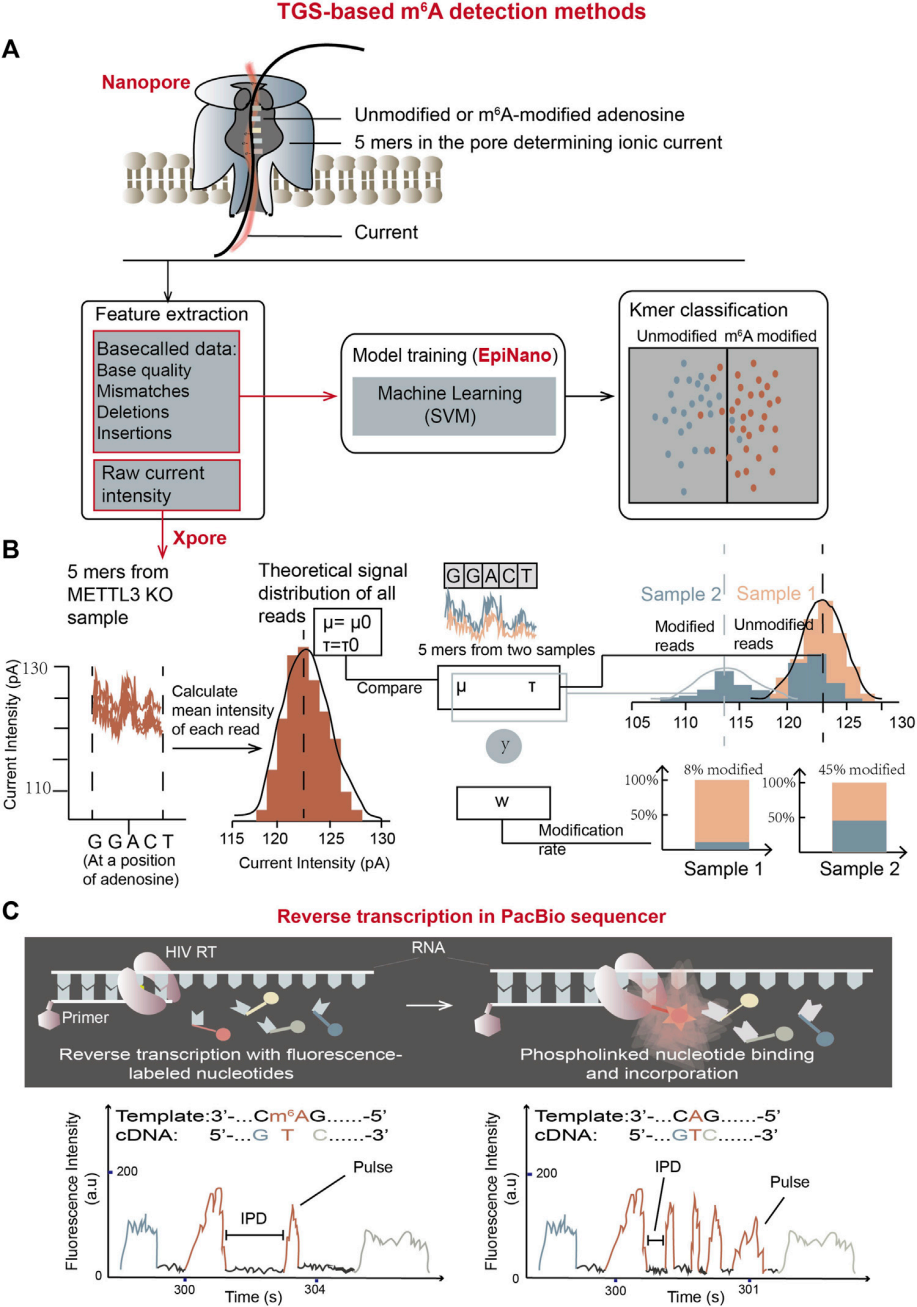
m^6^A detection methods using TGS platforms. **(A)** The schematic overview of m^6^A detection using Oxford direct RNA sequencing (DRS) combined with “base call error rate”-based EpiNano algorithm. The trained model is able to classify bases in an undetermined RNA sample into m^6^A-modified group and unmodified group. **(B)** The schematic workflow of m^6^A quantification using Oxford DRS combined with xPore algorithm which is based on raw current signals (Pratanwanich et al., 2021). In the graph, y represents input (5-mer reads), and w represents the weight of the modified reads at said location. In detail, 92% of reads in sample-1 (orange sample) belong to the theoretical Gaussian distribution, meaning the modification rate of sample-1 at the location is 8%; while the modification rate of sample-2 (blue sample) at the location is 45%. **(C)** Schematic representation of m^6^A detection using PacBio SMRT reverse transcription (Vilfan et al., 2013). Two movies showed below represent the trace of reverse transcription from a m^6^A-containing template (left) and an unmodified template (right). Compared with unmodified residues, the pulse frequency is reduced while IPD is elevated in m^6^A-modified residues, which can work as features to determine m^6^A modifications in unknown samples.

Epinano (Liu et al., 2021), a supervised learning algorithm, aims at using base call error features to train the support vector machine (SVM)-based classifier and predicting the m^6^A (Figure 9A). The training data were from two sets of in vitro-transcribed RNAs, which comprised all possible 5-mer sequences with m^6^A-modified or unmodified adenosines. Three features, base quality, deletion frequency and mismatch frequency from m^6^A-modified and unmodified data sets were used to train the SVM model (Figure 9A). The trained model could then be used for prediction of m^6^A at the positions of RRACH motifs (Liu et al., 2019; Smith et al., 2020; Liu et al., 2021).

DRUMMER, Eligos2, JACUSA2, and DiffErr algorithms mainly rely on significantly different error profiles between two sets of RNAs with m^6^A or without m^6^A (Parker et al., 2020; Price et al., 2020; Jenjaroenpun et al., 2021; Piechotta et al., 2022). Two comparative sets of RNAs could come from either *in vitro* transcription or *in vivo* impairment of the relevant gene functions. The m^6^A locations are determined through multiple statistical computation of insertions, deletions and substitutions between two conditions in these algorithms.

#### 4.1.2 Algorithms based on raw ionic current signals

Nanocompore, xPore, Tombo, m^6^Anet, MINES, nanom^6^A, Yanocomp, and DENA are algorithms developed to detect m^6^A by analyzing the raw current signals (Lorenz et al., 2020; Gao et al., 2021; Leger et al., 2021; Pratanwanich et al., 2021; Abebe et al., 2022; Qin et al., 2022). The raw signals are dissected into “events” and assigned to their corresponding nucleotides. Further ionic flow features such as current intensity and dwell time in the raw signals are exacted and employed to train a model or implement comparative analyses based on statistical tools in these algorithms.

In 2021, xPore (Pratanwanich et al., 2021), an algorithm to measure m^6^A quantitatively in multiple samples using raw current signal intensity as a feature was developed (Figure 9B). The assumption in xPore is that when modeling m^6^A modification states at a single genomic locus, raw current signal intensity should have two Gaussian distributions corresponding to unmodified and modified RNA species. These two distributions could be shared across different samples and allow individual reads from the genomic locus to fit both distributions with different degrees. With this assumption, xPore models current intensity from multiple samples by two Gaussian Mixture Model (GMM), which is a form of supervised learning (Pratanwanich et al., 2021). The algorithm requires prior information about the theoretical signal distribution of unmodified RNA species to guide model development. For each particular location of 5-mer nucleotides with an adenosine in the middle, intensity-level mean of each read is calculated first. Then, the distributions and standard deviations of the mean intensity from all reads covering the particular position are computed for different samples by GMM to separate mean intensity from all reads into two gaussian distributions corresponding to modified and unmodified adenosine-containing sites. m^6^A rates at each location for different samples could then be calculated by read numbers in the two distributions. xPore can accurately quantify RNA modifications at the positions with high modification rates (higher than 25%).

Nanocompore was developed in which both current intensity and dwell time features are used to cluster signals from m^6^A modified and unmodified RNA species with a univariate pairwise test or a bivariate classification method based on two-component GMM clustering (Leger et al., 2021). The following logistic regression test determines whether the distributions of two clusters are significantly different. Using these grouping methods, Nanocompore is able to conduct m^6^A calls by comparing an undetermined RNA sample with an unmodified control sample.

One great advantage of these nanopore DRS methods is their straight forward procedure. These methods don’t need complex experimental signal transformation processes or special treatment of mRNA molecules. Nanopore DRS raw data can maintain the original RNA modification information. Besides, they analyze entire mRNA molecules, which allows to observe m^6^A at specifically-spliced isoforms and correlate m^6^A status with other transcript features. However, these methods require extensive iterative sequencing signal measurement to calculate the error frequency in calling RNA sequences, which makes them inaccurate in detecting m^6^A sites with low modification rates. Proper control groups, improved algorithms, new nanopore proteins specially engineered for m^6^A detection, and large simulations are required to improve the accuracy and sensitivity of the currently-used methods.

### 4.2 The m^6^A detection using PacBio SMRT reverse transcription

PacBio platform employs sequencing by synthesis (SBS) using fluorescence-labelled dNTP, and invents a new method called zero mode waveguides (ZMWs) to detect fluorescent signals emitted only from the area of single molecular DNA synthesis. The ZMW provides a highly confined optical observation volume, enabling single-molecule-resolved biophysical studies in the existence of other fluorescent molecules (Eid et al., 2009).

PacBio Single molecule Real Time (SMRT) DNA sequencing has been optimized to RNA sequencing by utilizing an HIV reverse transcriptase (HIV RT) to determine both RNA sequences and modifications simultaneously in PacBio sequencer (Vilfan et al., 2013). Similar to SMRT DNA sequencing (Eid et al., 2009), RT activity is visualized by phospho-linked deoxyribonucleotides, in which the terminal phosphates carry fluorophores. The fluorescent label is released during nucleotide incorporation so the cDNA synthesis can be detected in real time (Figure 9C). Furthermore, at RNA m^6^A sites, the complementary nucleotide incorporation displays the different kinetics compared with unmodified counterparts. Fluorescent pulse frequency at m^6^A locations is reduced compared with their unmodified RNA control locations, indicating that the binding of phospho-linked nucleotide is affected by m^6^A on template RNA. In addition, the interpulse durations (IPDs) at m^6^A-modified positions are increased than those at unmodified positions, meaning that the binding rates of complementary TTP at m^6^A sites are decreased by approximately 5-fold. Based on this principle, comparing the distributions of IPDs between two RNA species can reveal the precise locations of m^6^A (Vilfan et al., 2013).

Although this method has not reached the transcriptomic level, it has however, opened up the possibility for genome-wide m^6^A detection using PacBio SMRT reverse transcription.

## 5 Discussion

This comprehensive review presented major sequencing-independent m^6^A detection methods as well as SGS-based and TGS-based transcriptome-wide m^6^A detection methods. Since these methods carry distinct features, proper methods should be chosen considering the criteria such as the required starting materials, sensitivity, stoichiometry, site resolution, bias in detection, convenience of reagent and software, procedure simplicity, result reproducibility, and cost (Supplementary Table S1).

The sequencing-independent biochemical methods can achieve two goals: m^6^A-ELISA and LC/MS could quantify the m^6^A level in total mRNA; SCARLET and SELECT could investigate m^6^A status at individual sites (Figure 2; Supplementary Table S1). Among them, m^6^A-ELISA, LC/MS, and SCARLET have been extensively used. For rough quantification of total m^6^A levels, m^6^A-ELISA is a good choice because it is a simple, cheap, and commercially-available method (Figure 2C). This method is very suitable for comparison of total m^6^A levels in different samples using ∼5 μg of total RNA. Comparing with m^6^A-ELISA, LC/MS can more accurately quantify total m^6^A levels in samples with great sensitivity to the m^6^A level change using ∼50 μg of total RNA. Also, LC/MS can discriminate different nucleosides with/without varying modifications, so it will be good to quantify the differentiation in nucleoside compositions of total mRNAs from multiple samples. If individual candidate m^6^A sites are expected to have important function, SCARLET can precisely validate the modification status of the sites and quantify m^6^A and unmodified adenosine levels at the sites (Figure 2A). Anyway, SCARLET needs to use ∼1 μg of mRNA (>20 μg of total RNA) and ^32^P-radioisotope. If only a small amount of RNA is available, a radioisotope-free, qPCR-based method SELECT can be employed to quantify individual candidate m^6^A sites using as low as 0.2 ng of mRNA (Figure 2B).

To meet the increasing requirements for epitranscriptomic study, many high-throughput sequencing-based m^6^A profiling methods have been developed. So far, there are three main categories for these methods: 1) SGS-based anti-m^6^A antibody-dependent methods; 2) SGS-based anti-m^6^A antibody-independent methods, which rely on enzymes, SAM analogs, or chemicals; 3) TGS and machine learning-based methods.

In the first category, MeRIP and CLIP-based miCLIP, meCLIP and m^6^ACE-seq, all rely on m^6^A antibody (Supplementary Table S1). MeRIP and miCLIP are the most widely used methods so far and have revealed transcriptomic m^6^A modification for the first time. Also, CLIP-based miCLIP, meCLIP and m^6^ACE-seq have achieved the single-nucleotide resolution in m^6^A-site detection. However, the requirement in a large amount of mRNA (10–20 μg of mRNA for CLIP-based methods and 400 μg of mRNA for MeRIP) and the massive dependence on anti-m^6^A antibody cause major limitations (McIntyre et al., 2020). Due to low specificity of antibody, these methods usually exhibit high noise. Detection accuracy in these methods can be improved after calibration m^6^A signals by a negative control—a modification-free, endogenous transcriptome-resembling, synthetic RNA library (Zhang Z. et al., 2021). This provides a way to evaluate and calibrate some noise in the antibody-dependent m^6^A detection methods. Nevertheless, some anti-m^6^A antibodies may react with m^6^Am dimethyl adenosine as well, which will make these methods hardly distinguish the two modifications (Schwartz et al., 2013; Schwartz et al., 2014a). More importantly, the antibody-based methods are not ideal for accurate m^6^A quantification, which is fundamental in addressing critical questions about m^6^A cellular function and its response to environmental stimuli (Meyer and Jaffrey, 2014; Schwartz, 2016; Grozhik and Jaffrey, 2018). Therefore, new methods have been emerged.

The methods in the second category are based on enzymes, SAM analogs, or chemicals. DART-seq and Mazter-seq/REF-seq can map m^6^A sites at single-base resolution, while others such as m^6^A-SAC-seq, eTAM-seq, and GLORI can achieve stoichiometric measurement at each m^6^A site in addition to single-base resolution. Specially, scDART-seq can achieve single-cell m^6^A profiling, which is necessary for highly-heterogeneous tissues and is critical for identification of cell-specific m^6^A patterns and roles. Furthermore, long-read DART-seq using PacBio platform can determine m^6^A sites transcript-isoform specifically. Considering simple procedure and low cost, the recently-developed GLORI and eTAM-seq have good potential, especially if commercial TadA8.20 enzyme is available in eTAM-seq (Supplementary Table S1). However, among these new methods, Mazter-seq/REF-seq can only detect m^6^A sites with an ACA motif, accounting for 16%–25% of total m^6^A sites. Also, DART-seq relies on the transfection efficiency of cells and accompanies an over-expression problem. In addition, m^6^A-SAC-seq exhibits a bias of m^6^A detection towards the GAC sites, and its quantification relies on a standard curve from spike-in RNA. In summary, these new methods give great opportunity to illustrate new biological phenomena in the field.

The methods in the third category are based on TGS and machine learning. This developing area provides new opportunity. Nanopore DRS-based methods are able to collect signals directly from original RNA molecules, retaining untransformed modification information (Supplementary Table S1). Further, the simple library construction makes TGS-based methods time saving and convenient to use. Also, they have achieved the single-nucleotide resolution, and Nonocompore even provides mRNA isoform-specific detection. However, they have limitations in high cost, low accuracy, and high requirements for RNA quality. For example, the quantitation results of xPore are not in accordance with other methods like REF-seq (Zhang et al., 2019), which may be raised from the variance of the same 5 mers’ current intensity. Besides, the machine learning algorithms used for model training may not be really suitable for m^6^A prediction. Thus, TGS-based methods solicit further improvement both in computational algorithms and experimental techniques such as RT enzyme, motor/nanopore protein and library construction.

In summary, current technology has facilitated significant advancement in m^6^A research, and it will be useful to combine the transcriptomic methods with the site-specific quantification methods like SCARLET or SELECT. Since most SGS-based methods, including the latest eTAM and GLORI, need *in vitro* fragmentation and antibody, enzymatic or chemical treatments before sequencing library construction, isoform-specific m^6^A information is lost. Therefore, it is necessary to put effort into developing mRNA isoform-specific m^6^A profiling methods and three-dimensional m^6^A pattern profiling methods. Anyway, the methods introduced here can meet varying requirements of m^6^A research, and will provide important insights for developing new strategies in different fields.
