# Supplementary material for: Current progress in strategies to profile transcriptomic m6A modifications
Source: Front Cell Dev Biol. 2024 Jul 11;12:1392159. doi: 10.3389/fcell.2024.1392159 (PMC11269109; doi:10.3389/fcell.2024.1392159)
Supplement: Supplementary file 1 [file Table1.pdf]

Supplementary Table 1| Comparison of m^6A Measuring Methods

Technique	Description	Input amount	Resolution	Pros	Cons
**Sequencing-independent methods**					
m^6A-ELISA	The m^6A-ELISA kit can detect the absolute or relative amounts of m^6A using m^6A antibody.	25 ng mRNA/ 5 µg total RNA	per sample	> Commercially available kit<br>> Cost-saving and sensitive<br>> Adaptable to other modifications	> Standard curve needed<br>> Lacking location information
LC/MS	Poly(A)-tailed RNAs are hydrolyzed into single nucleoside; these nucleosides are analyzed by LC/MS, which detects and quantifies m^6A and other nucleosides according to mass-to-charge ratios.	50 µg total RNA	Total m^6A level per sample	> Accurate<br>> Sensitive<br>> Easy sample preparation	> Expensive<br>> No transcript and site location Information<br>> Large input amount requirement
SCARLET	An m^6A site of interest is labeled with radioisotope ^{32}P after site-specific cleavage using RNase H and complementary chimeric oligoes. The labeled mono-m^6A and mono-adenosine from the desired site are finally separated, visualized, and quantified using TLC.	1 µg mRNA	1 nt	> Accurate<br>> Sensitive<br>> Quantification	> Radioisotope usage<br>> Time-consuming<br>> Laborious protocol<br>> Low throughput: a single site
SELECT	The up and down DNA probes are annealed to mRNA but leave an unpaired 1-nt gap at the site opposite the m^6A of interest. DNA elongation and nick ligation are hindered by m^6A, less qPCR products are formed from m^6A than unmodified-A.	0.2 ng mRNA	1 nt	> Sensitive<br>> Low input amount<br>> Easy protocol<br>> Flexible	> Standard RNA mixture needed<br>> Standard curve needed<br>> Low throughput: a single site
**SGS-based methods**					

SGS-based anti-m^{6}A antibody-dependent methods					
meRIP	RNA IP	400 µg mRNA	100-200 nt	> Simple procedure<br>> Widely used<br>> Transcriptomic mapping	> Low resolution<br>> High noise
miCLIP	RNA IP, radioactive label, gel purification	~20 µg mRNA	1 nt	> Good accuracy<br>> Widely used<br>> Low sequence bias in detection<br>> Transcriptomic mapping	> Complicated<br>> Radioisotope usage<br>> Low to medium noise<br>> Large input amount requirement
meCLIP	RNA IP, gel purification	~10 µg mRNA	1 nt	> Improved library complexity<br>> Free from radioisotope<br>> Transcriptomic mapping	> Low to medium noise<br>> Complicated and laborious
m^{6}ACE-seq	IP, XRN1 digestion,<br>synthesized m^{6}A RNA spike-ins	~10 µg mRNA	1 nt	> Simple procedure<br>> Semi-quantification<br>> Spike-ins for antibody efficiency calibration<br>> Transcriptomic mapping	> Limited sensitivity<br>> Limited accuracy
SGS-based anti-m^{6}A antibody-independent methods					
DART-m^{6}A-seq	Expression of YTH-APOBEC1 fusion protein in cells, m^{6}A quantification based on C-to-U editing percentage.	100 ng-1 µg total RNA	Nucleotides	> Sensitive<br>> Single-cell level<br>> Transcriptomic mapping	> Artifacts from overexpression<br>> Limited accuracy (off-target)
Mazter-seq	MazF cuts at 5' of ACA but not m^{6}ACA, pile up the cut reads to find the ratio of ACA-starting reads to all reads.	100 ng mRNA	1 nt	> Stoichiometry<br>> High sensitivity<br>> Transcriptomic mapping	> Context bias in site detection<br>> Lack of absolute quantification controls<br>> Quantification is insert length distribution dependent

m^6A-SEAL-seq	FTO oxydation, DTT treatment,<br>biotin labeling, streptavidin enrichment	1 μg mRNA	50-200 nt	> No context bias<br>> Accurate<br>> Transcriptomic mapping	> Low resolution<br>> Low sensitive<br>> Time-consuming and laborious<br>> High cost
eTAM-seq	TadA8.20 deamination, m^6A spike-ins,<br>modification-free IVT controls	50 ng mRNA	1 nt	> No context bias<br>> Accurate<br>> Stoichiometry<br>> Sensitive<br>> Transcriptomic mapping	> Low sensitivity at the sites with low modification levels<br>> No commercial TadA8.20<br>> High cost
m^6A-SAC-seq	In vitro labeling m^6A by allyl-SAM<br>and dimethyltransferase MjDim1;<br>Iodine treatment; high mutation rate at<br>m^6A site in RT	2-50 ng<br>mRNA	1 nt	> High sensitivity<br>> High accuracy<br>> Transcriptomic mapping<br>> Stoichiometric	> Complicated procedure<br>> No commercial MjDim1<br>> No commercial SAM analog<br>> Context bias in site detection<br>> Costly
m^6A-label-seq	Labeling m^6A by allyl-SAM/allyl-SeAM in cell,<br>enrichment of labeled RNA with i^6A antibody,<br>high mutation rate at m^6A sites in RT	20 μg in vivo<br>labeled mRNA	1 nt	> Transcriptomic mapping<br>> The m^6A clusters are more easily detected than the others	> Artifacts from allyl-SAM inside cells<br>> Large input amount required<br>> No site stoichiometric information
GLORI	Glyoxal G protection,<br>Nitrite-mediated A deamination	100 ng mRNA	1 nt	> High accuracy<br>> High sensitivity<br>> Economic<br>> Stoichiometry<br>> No context bias<br>> Transcriptomic mapping	> Possible RNA degradation

**TGS-based methods**					
Epinano	Nanopore DRS base call error feature, SVM algorithm	500 ng mRNA	1 nt	> Easy protocol<br>> Transcriptomic mapping	> Limited accuracy<br>> No stoichiometric information<br>> Detectable context limited to "RRACH" motif
Xpore	Nanopore DRS raw signal intensity feature, GMM algorithm	100 ng mRNA	1 nt	> Stoichiometry measurement<br>> No context bias<br>> Easy protocol<br>> Transcriptomic mapping	> Limited accuracy
Nanocompore	Nanopore DRS raw signal intensity and dwell time features; GMM algorithm	100 ng mRNA	1 nt	> One-click pipeline available<br>> Statistically reliable<br>> Isoform specific<br>> Transcriptomic mapping	> Limited accuracy<br>> Unmodified control sample need<br>> No stoichiometric information
Pacbio DRS	RT using HIV reverse transcriptase in PacBio sequencer	-	1 nt	> High resolution	> Limited accuracy<br>> Commercially unavailable<br>> High cost<br>> No stoichiometric information<br>> Not a transcriptomic level
